# Luminescence studies on green emitting InGaN/GaN MQWs implanted with nitrogen

**DOI:** 10.1038/srep09703

**Published:** 2015-04-08

**Authors:** Marco A. Sousa, Teresa C. Esteves, Nabiha Ben Sedrine, Joana Rodrigues, Márcio B. Lourenço, Andrés Redondo-Cubero, Eduardo Alves, Kevin P. O'Donnell, Michal Bockowski, Christian Wetzel, Maria R. Correia, Katharina Lorenz, Teresa Monteiro

**Affiliations:** 1 Departamento de Física e I3N, Universidade de Aveiro, Campus Universitário de Santiago, 3810-193 Aveiro, Portugal; 2 IPFN, Instituto Superior Técnico, Campus Tecnológico e Nuclear, E.N. 10, P-2695-066 Bobadela LRS, Portugal; 3 Departamento Física Aplicada, Universidad Autónoma de Madrid, 28049 Madrid, Spain; 4 SUPA Department of Physics, University of Strathclyde, Glasgow, G4 0NG, Scotland, UK; 5 Institute of High Pressure Physics, Polish Academy of Sciences, 01-142 Warsaw, Poland; 6 Department of Physics and Future Chips Constellation, Rensselaer Polytechnic Institute, Troy, New York 12180, USA

## Abstract

We studied the optical properties of metalorganic chemical vapour deposited (MOCVD) InGaN/GaN multiple quantum wells (MQW) subjected to nitrogen (N) implantation and post-growth annealing treatments. The optical characterization was carried out by means of temperature and excitation density-dependent steady state photoluminescence (PL) spectroscopy, supplemented by room temperature PL excitation (PLE) and PL lifetime (PLL) measurements. The as-grown and as-implanted samples were found to exhibit a single green emission band attributed to localized excitons in the QW, although the N implantation leads to a strong reduction of the PL intensity. The green band was found to be surprisingly stable on annealing up to 1400°C. A broad blue band dominates the low temperature PL after thermal annealing in both samples. This band is more intense for the implanted sample, suggesting that defects generated by N implantation, likely related to the diffusion/segregation of indium (In), have been optically activated by the thermal treatment.

InGaN/GaN multi-quantum wells (MQW), used in light emitting diodes (LEDs), are relevant to a broad range of applications in communications, sensing and lighting. Even at low injection levels, longer-wavelength (e.g. green) LEDs exhibit reduced internal quantum efficiency (IQE)^1^. Low IQE and a general drop of efficiency at high injection currents are attributed to polarization-induced electric fields that lead to a reduction of the integral overlap of electrons and holes wave functions (quantum confined Stark effect, QCSE) and losses due to the Auger effect^2^. Both effects are especially strong in green LEDs due to their high InN content and InGaN resonance in the band structure^2^,^3^. In order to avoid non-radiative processes, the promotion of the quantum well intermixing (QWI) by using ion implantation and annealing was proposed^3^,^4^. The ion beam generates vacancies and interstitials within a penetration depth controlled by the selected beam energy. Subsequent thermal annealing promotes the recovery of lattice damage as well as defect diffusion through the structure. The expected change in the shape of the QW should alter the band structure^5^. Theoretical studies suggest a strong increase in IQE for a quasi-parabolic gradient of composition by interdiffusion of elements between the QW and the barrier layers^3^. For this reason, the role of the ion implantation effects and heat treatments on the structural and optical properties of the MQW should be thoroughly investigated.

In this work we analyse the effects of N implantation and annealing at high temperature and high pressure (HTHP) on the optical properties of green-emitting InGaN/GaN MQW. The investigation is conducted by using temperature dependent and excitation power dependent photoluminescence (PL), PL excitation (PLE) and decay times measurements. The changes in the optical spectra of the InGaN/GaN MQW structures promoted by the post-growth treatments will be analysed and discussed and models for the recombination processes will be established.

## Experimental procedure

The samples used in this study were grown on *c*-plane oriented sapphire substrate by metal organic chemical vapour deposition (MOCVD) as described elsewhere^6^. The InGaN/GaN MQW structure was grown on a 5 μm-thick GaN layer on (0001) sapphire substrate and capped by a 20 nm thick GaN layer. The target structure consists of a ten-period MQW with ~2 nm InGaN QW and ~20 nm-thick GaN barriers. The average InN content in the QW was determined to be 10% by Rutherford Backscattering Spectrometry.

In this study we exposed four identical samples to different treatments. One, the as-grown sample was kept as a reference of the virgin material (hereafter labelled #as-grown). Two of the samples were simultaneously implanted with N to a total fluence of 7 × 10^13^ cm^−2^ using three different energies of 35, 80 and 160 keV aiming to produce a homogeneous defect profile throughout the MQW region. The implantation was carried out at room temperature (RT) off the *c*-axis to avoid possible channelling effects. One implanted sample was kept as a reference and labelled #as-imp. After the implantations, we performed a HTHP annealing at 1400°C in a 1.1 GPa N_2_ atmosphere for 30 min on two samples: one of the as-implanted (to produce #as-imp-HTHP) and one of the as-grown (to produce #as-grown-HTHP). The conditions for the annealing were chosen in light of previous optimizations on test samples, where rapid thermal annealing up to 1000°C and HTHP annealing at 1250°C did not show significant changes in the optical properties (to be published elsewhere).

Steady state photoluminescence (PL) spectroscopy was performed as a function of temperature (from 14 K to RT) using a cold finger He cryostat. The 325 nm line of a cw He-Cd laser (power density I_0_ < 0.6 W.cm^−2^) was used as excitation source. The sample luminescence was dispersed by a SPEX 1704 monochromator (1 m, 1200 gr.mm^−1^) and detected by a cooled Hamamatsu R928 photomultiplier. Photoluminescence excitation (PLE) and PL spectra were recorded at RT using a Fluorolog-3 Horiba Scientific modular apparatus with a double additive grating scanning monochromator (2 × 180 mm, 1200 gr.mm^−1^) in the excitation channel and a triple grating iHR550 spectrograph (550 mm, 1200 gr.mm^−1^) coupled to a R928 Hamamatsu photomultiplier for emission. A 450 W Xe lamp was used as excitation source. The measurements were carried out using a front face acquisition mode, and the presented spectra were corrected for the spectral responses of the optical components and the Xe lamp. RT lifetime measurements were acquired with the same Fluorolog-3 system using a NanoLED-370 (1.3 ns pulse duration) as excitation source and the DataStation software for the data analysis.

## Results and Discussion

### As-grown sample

**Figure 1:**
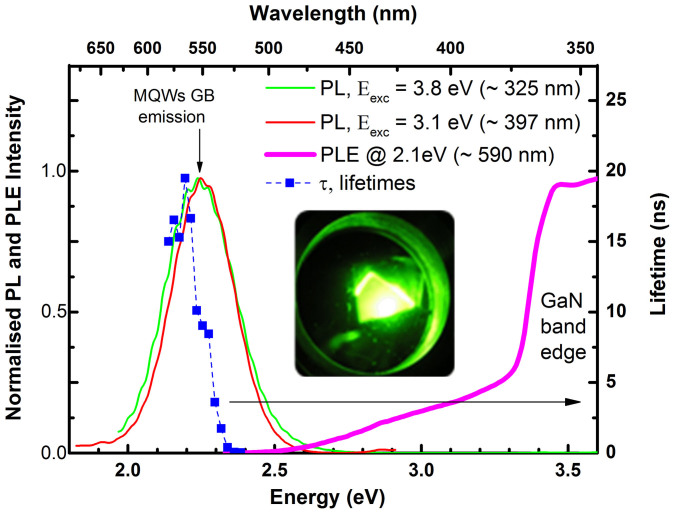
RT normalized PL (obtained under 3.8 eV and 3.1 eV excitations) and PLE spectra (monitored at 2.1 eV; 590 nm) to the unit peak height of the #as-grown sample. Inset: photograph of the low temperature emission. Energy dependent PL decay measurements obtained with 3.36 eV (λ_exc_ = 370 nm) nanoLED pulsed excitation.

Figure 1 (a) shows the RT PL and PLE spectra at RT and the visual appearance (in inset) of the as-grown InGaN/GaN MQW sample under He-Cd excitation at 14 K. The green band (GB) emission peak position, near 2.3 eV, was found to be slightly dependent on the measured spot, likely due to indium nitride compositional heterogeneities in the alloy and/or well width fluctuations^7^,^8^,^9^. The full width at half maximum of the GB PL at RT (~180 meV) is in agreement with those reported for similar structures^6^. The PLE spectrum indicates that GB emission may be achieved by pumping the samples both above and below the GaN bandgap; a wide excitation band with an onset at ~2.5 eV precedes a steep increase at the GaN absorption edge. Pumping the ternary alloy directly, with excitation below the GaN band edge reproduces the emission band nearly exactly, suggesting that the lower energy excitation band corresponds to local MQW–related absorption. The broadening of the excitation band quantifies the spatial composition fluctuations in the InGaN/GaN structure^10^,^11^. The PL broad emission correlated with compositional and well width fluctuations identified by transmission electron microscopy in the MQW samples (to be published elsewhere) agrees well with the localized excitons model for the recombination processes.

We also analysed the RT GB emission kinetics by exciting below GaN bandgap, to probe the carrier dynamics of the QW emission at different energies (Figure 1). Rather than to a single exponential decay, typical for un-localized excitons, the experimental PL decays were best fitted to a stretched exponential model (*I*_0_ exp(−*t*/*τ*)*^β^*), where τ corresponds to the mean decay time, and β to the dispersion factor (0 < β < 1). The stretched exponential decay behaviour is often encountered in systems with disorder and is considered to be a result of diffusion of excited carriers^12^,^13^,^14^,^15^. Some of the best-fit PL decay lifetimes τ and β values are summarized in Table 1.

The PL lifetimes τ were found to be within the typical range for the decay of localized excitons in InGaN/GaN MQW structures^16^,^17^. In addition, the PL decay times, τ, are found to depend on the emission detection energy, and they are constant through the lower half of the band, consistent with the existence of a 2D mobility edge^18^. A value of β lower than 1 is related to the increase of spatial separation of electrons and holes, temporarily suppressing radiative recombination. As we approach the maximum of the emission band, β seems to increase, indicating that the emission comes from more strongly localized states. The physical reasons for that could be the excitation of carriers from localized to extended states, multiple trapping-detrapping or hopping between localized states^13^.

The intensity of the green band decreases with increasing temperature between 14 K and RT. This thermal quenching is well fitted to a classical model with two nonradiative competitive channels^19^
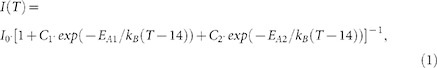
where *I_0_* is the intensity at 14 K, *C*_1,2_ temperature independent effective degeneracies, *k_B_* the Boltzmann constant and *T* the absolute temperature. The best fit to the experimental data yields activation energies of *E_A_*_1_ = 5.3 ± 0.9 *meV* and *E_A_*_2_ = 43.5 ± 6.6 *meV* with pre-exponential factors of *C*_1_ = 1.8 ± 0.4 and *C*_2_ = 23.8 ± 7.3, respectively, as shown in Table 2 for the #as-grown sample. The two activation energies describing the thermal quenching of the MQW GB emission correspond to delocalization of excitons from potential fluctuations and thermal excitation from confined energetic states to the continuum, respectively, as previously reported^19^.

### Effects of post-growth treatments

**Figure 2:**
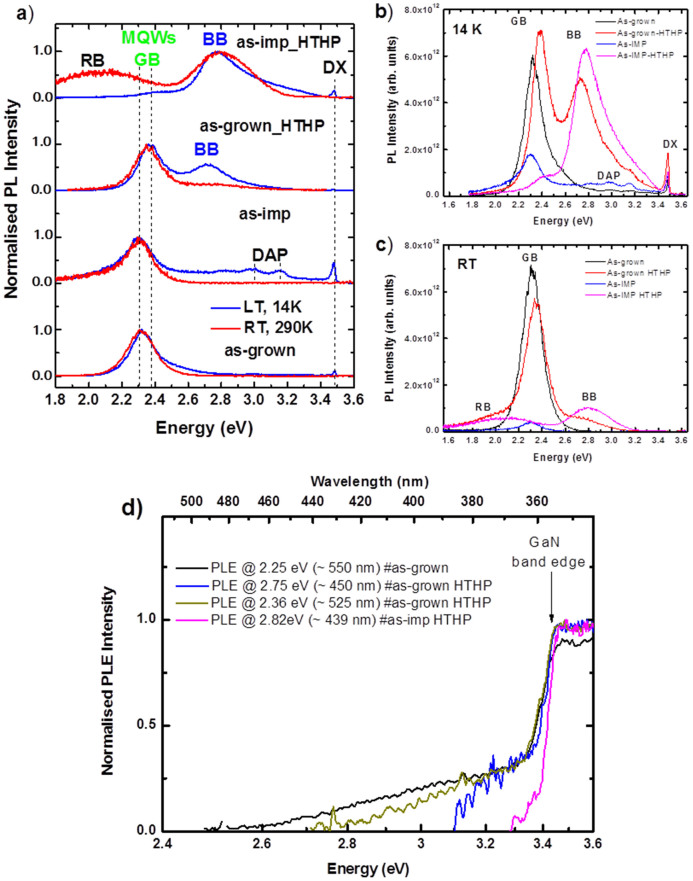
(a) Normalized PL spectra to the unit peak height for the #as-grown, #as-grown-HTHP, #as-imp and #as-imp-HTHP samples at 14 K and RT obtained with 3.8 eV excitation. The spectra are vertically shifted for clarity. PL spectra of the samples obtained at 14 K (b) and RT (c) under 3.8 eV excitation showing the variation of the PL intensity with the implantation and annealing post-growth treatments. (d) RT normalized PLE spectra (monitored at PL peak maxima) to the unit peak height of the #as-grown, #as-grown-HTHP, and #as-imp-HTHP samples.

Figure 2 (a) compares normalized PL spectra at 14 K and RT for all 4 samples, #as-grown, #as-imp, #as-grown-HTHP and #as-imp-HTHP. The as-grown and as-implanted samples exhibit a single MQW GB emission; after annealing, however, both show the presence of an additional blue band (BB). The GB is clearly affected by N implantation and thermal annealing treatments. Indeed, after N implantation, a strong reduction of the 2.3 eV GB intensity is observed (Figures 2b) and 2c)). However, if we compare the GB observed for the #as-grown and #as-imp samples, no changes are seen in the peak position or the spectral shape. This indicates that the damage induced by the N implantation decreases the MQW luminescence without promoting new optically active defects. In contrast, HTHP thermal annealing generates new optically active centres in both cases: an unstructured BB appears with peak around 2.7–2.8 eV in the #as-grown-HTHP and #as-imp-HTHP samples. In addition, a significant change is observed for the MQW GB emission. For the #as-grown- HTHP sample, the GB emission peak blue-shifts by about 100 meV, compared to the #as-grown sample, this might be related to indium interdiffusion to the GaN barrier region. GB recombination in the #as-imp-HTHP sample appears almost totally suppressed; in any case the fate of the GB is obscured by the rise of a new band, labelled RB in Figures 2a) and 2c). The preferential excitation pathways of the optical GB and BB were identified via RT PLE as shown in Figure 2d). The spectra were monitored at PL peak maxima 550 nm, 525 nm and 450 nm, and 439 nm for the #as-grown, #as-grown-HTHP, and #as-imp-HTHP samples, respectively. Due to the high damage of the #as-imp sample, no RT PLE signal could be recorded. In all the cases the GB and BB could be excited via above and below the GaN bandgap. However the blue shift observed on the onset of absorption indicates that a decrease of the indium nitride content upon annealing might have taken place.

**Figure 3:**
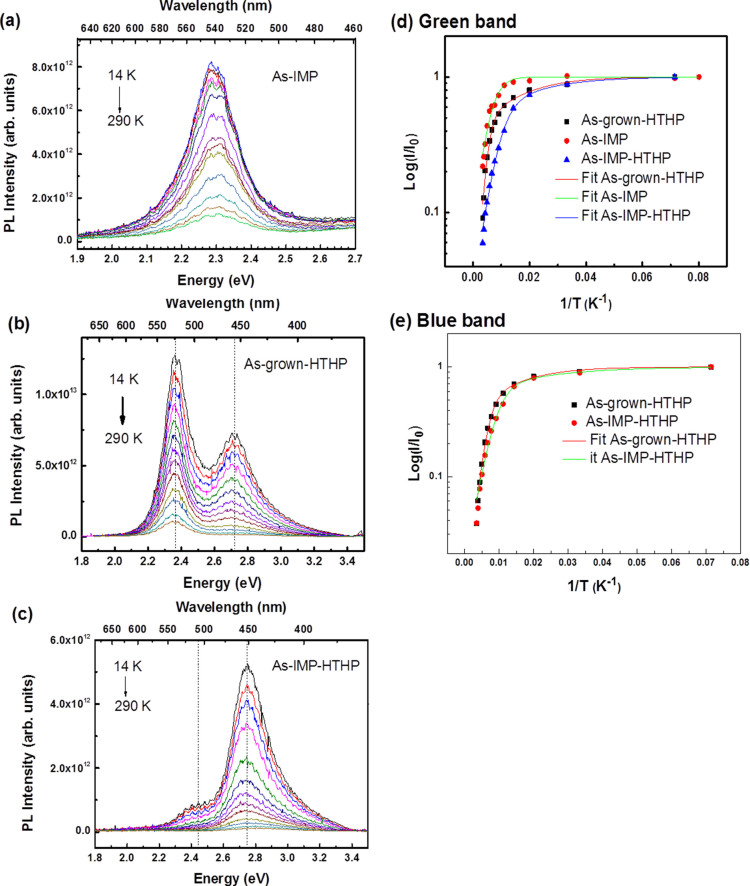
Temperature dependent PL spectra obtained with a 3.8 eV photon excitation for samples: (a) #as-imp; (b) #as-grown-HTHP; (c) #as-imp-HTHP. (d) and (e) integrated intensity dependence of the green and blue bands as a function of 1/T. Full lines correspond to the best-fit to the experimental data according to eq. 1 using the parameters summarized in Table 2.

Temperature-dependent PL spectra of the bands observed in the #as-imp (green only), #as-grown-HTHP and #as-imp-HTHP samples (green and blue), are presented in Figures 3 (a), (b) and (c), respectively. The temperature dependences for the #as-grown, #as-imp and #as-grown-HTHP samples reveal no significant peak shifts, but a general tendency of intensity decrease with increasing temperature is observed for all the bands. Thermally activated nonradiative pathways are well described by the activation energies presented in Table 2 derived from fitting equation (1) to the experimental data. Although the best fits were achieved considering two activation energies with similar values for the #as-grown and #as-grown-HTHP samples, a single activation energy yields a good fit for the #as-imp sample (Figures 3 (d) and (e) for GB and BB, respectively). In the latter case, where a higher defect concentration with respect to the #as-grown sample is expected due to implantation damage, the absence of the small activation energy is related to distinct carrier de-trapping mechanisms for the localized excitons, yielding a PL thermal quenching assisted via different relaxation processes.

**Figure 4:**
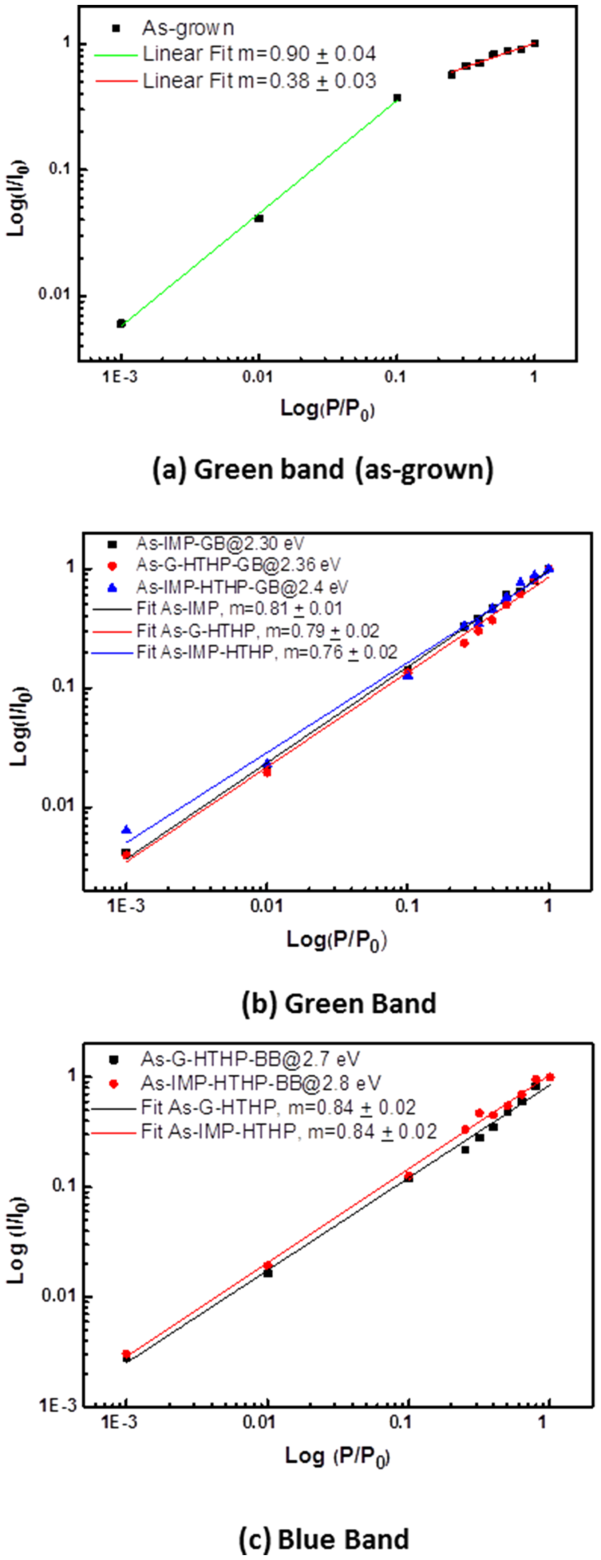
Integrated PL intensity dependence on the excitation intensity for the green band in the #as-grown sample (a), and green (b) and blue (c) in the post growth treated samples. Full lines correspond to the best fits of the experimental data according with a power law dependence (*IαP^m^*).

The appearance after HTHP annealing of a blue band, actually the dominant recombination in the #as-imp-HTHP sample, (Figure 3c)), deserves to be explored more deeply. Blue bands have been extensively studied in unintentionally and intentionally doped GaN^20^. In the case of non-intentionally doped GaN layers, such as those involved in our MQW samples, a 2.9 eV blue luminescence has been reported^20^,^21^,^22^. This luminescence behaves like the one of a donor-acceptor pair (DAP) at low temperatures, transforming to a free-to-bound (e-A) recombination at temperatures above ~100 K^21^. Moreover the emission band exhibits a full width at half maximum (FWHM) of ~400 meV, decay times in the microsecond range, and a luminescence thermal quenching for temperatures above 200 K with an activation energy of 380 meV^21^,^22^. As shown in Figure 3, the BB identified in the present MQW structures has a narrower FWHM, a thermal quenching described by two relatively small activation energies (Table 2) and shows a RT lifetime shorter than 1 ns, since no measurable signal could be collected by using the current experimental set-up. These evidences clearly indicate that the BB in the studied samples behaves very differently from that previously reported in undoped GaN layers^20^,^21^,^22^ both as a function of temperature (Figure 3) and excitation density (Figure 4). Furthermore, the PLE monitored at the BB band have shown unequivocally the changes on the onset of the absorption, well below the GaN near band edge, which is much more consistent with the hypothesis to relate the observed BB emission to defects in the InGaN active layers or InGaN/GaN interface regions, specifically InN-poor regions as identified by TEM after HTHP annealing (to be published elsewhere). In order to identify a recombination model for the luminescence bands in our samples, further PL studies were realized as a function of excitation density, as presented in Figure 4. No power-dependent shift of either band maximum was observed, discounting any recombination model involving DAP transitions. As a function of the excitation density, the PL intensity can be well fitted to a power law over three decades of excitation density, *IαP^m^* with an exponent close to unity (Figures 4 (a), (b) and (c)). T. Schmidt *et al.* reported a PL power dependence well described by an exponent *m* between 1 < *m* < 2 for free and bound excitons^23^. A deviation of the power law was observed for higher decades, namely when saturation effects occur^23^. As shown in Figure 4 (a), at high excitation densities, the green band intensity for the #as-grown sample saturates as a consequence of the limiting radiative decay process. On the contrary, the same GB power dependence analysis for the #as-imp and #as-grown-HTHP samples do not exhibit such saturation. Furthermore, a similar linear behavior was found for the blue bands for both the #as-grown-HTHP and the #as-imp-HTHP samples, supporting a recombination model where the defects generated by post-growth treatments, such as those related with In redistribution^24^, are able to localize excitons.

## Conclusions

As-grown green emitting InGaN/GaN MQW were implanted with N ions and subjected to HTHP thermal annealing. A strong reduction of the QW green band PL intensity occurs upon implantation but the emission from the localized excitons is still observed without any change of the spectral shape or peak position. Nevertheless, the analysis of thermal stability of the green luminescence in the implanted sample shows that distinct nonradiative competitive channels occur for the as-grown and as-implanted samples, likely due to the influence of additional defects generated by the implantation. In this sample, the emission was well described by a single activation energy for the nonradiative processes, while in the as-grown sample two activation energies were necessary for the description of the luminescence de-excitation pathways. The dependence of the green band PL intensity on the excitation density exhibits a different behaviour in the as-grown and as-implanted samples. In particular, saturation effects were found to occur in the as-grown MQW structure with higher crystalline quality. Thermal annealing treatments at HTHP generates unstructured broad blue bands, also of excitonic nature, as suggested by the temperature and excitation density dependence of the luminescence intensity, likely to be related to the redistribution of In. Concerning implantation assisted QWI, it was not possible to recover the QW GB emission after implantation by thermal annealing suggesting that lower fluences should be employed to keep implantation damage low.

## Author Contributions

All the authors have made substantial intellectual contributions to the research work. This work was made in collaboration with different institutions responsible for the growth, implantation, annealing and characterization of the samples. These samples were subject to extensive structural and optical charactarizations by all the partners involved. C.W. was responsible for the growth of the analysed samples. M.B.L., E.A., K.L. and A.R.-C. performed the implantation and annealing treatments and carried the experiments and data analysis of the structural samples characterization. M.B. was responsible for the HPHT thermal annealing treatments. K.P.O'D. performed cathodeluminescence experiments. M.R.C. and T.C.E. were responsible for the Raman characterization and the optical measurements in the infrared spectral region. N.B.S. and J.R. carried out PLE and lifetime measurements and M.A.S. and T.M. performed temperature, excitation density and excitation energy dependent PL. All the authors have together discussed and interpreted the results. All the authors read and approved the final manuscript.

## Figures and Tables

**Table 1:** Best-fit PL decay lifetimes τ and β values using the stretched exponential model

Photon Energy (eV)	τ (ns)	β
2.34	0.40 ± 0.14	0.242 ± 0.001
2.29	3.60 ± 0.14	0.309 ± 0.002
2.25	9.03 ± 0.18	0.347 ± 0.001
2.21	16.64 ± 0.21	0.376 ± 0.001
2.19	19.50 ± 0.30	0.387 ± 0.001
2.17	15.30 ± 0.31	0.360 ± 0.001
2.14	15.00 ± 0.41	0.345 ± 0.002

**Table 2:** Activation energies and pre-exponential factors obtained from the temperature dependence of the Green and Blue bands by using a classical model (eq. (1))

	Green band (GB ~ 2.3 eV)				
	Ea_1_ (meV)	C_1_	Ea_2_ (meV)	C_2_	
#as-grown	5.3 ± 0.9	1.8 ± 0.4	43.5 ± 6.6	23.8 ± 7.3
#as-imp	–	–	33.2 ± 3.0	10.3 ± 2.2
#as-grown-HTHP	7.2 ± 0.9	1.5 ± 0.3	62.9 ± 7.6	81 ± 33
#as-imp-HTHP	5.7 ± 0.9	1.1 ± 0.3	29.3 ± 0.2	36.5 ± 4.8

	Blue band (BB ~ 2.7 eV)				
#as-grown	–	–	–	–
#as-imp	–	–	–	–
#as-grown-HTHP	8.4 ± 1.1	1.7 ± 0.4	55.3 ± 5.0	139 ± 45
#as-imp-HTHP	5.1 ± 1.2	0.8 ± 0.3	35.3 ± 2.8	60 ± 13

## References

[b1] Cho J., Schubert E. F. & Kim J. K. Efficiency droop in light-emitting diodes: challenges and countermeasures. Laser Phot. Rev. 7, 408–421 (2013).

[b2] Delaney K. T., Rinke P. & Van de Walle C. G. Auger recombination rates in nitrides from first principles. Appl. Phys. Lett. 94, 191109 (2009).

[b3] O'Donnell K. P., Auf der Maur M., Di Carlo A., Lorenz K. & SORBET consortium. . It's not easy being green: strategies for all-nitrides, all-colour solid state lighting. Phys. Stat. Sol. RRL 6, 49–52 (2012).

[b4] Redondo-Cubero A. *et al.* Selective ion-induced intermixing and damage in low-dimensional GaN/AlN quantum structures. Nanotechnol. 24, 505717 (2013). 10.1088/0957-4484/24/50/505717 24285147

[b5] Poole P. J. *et al.* The enhancement of quantum well intermixing through repeated ion implantation. Semicond. Sci. Technol. 9, 2134–2137 (1994).

[b6] Chen J.-H. *et al.* Optical and structural properties of InGaN/GaN multiple quantum well structure grown by metalorganic chemical vapor deposition. Thin Sol. Films 498, 123–127 (2006).

[b7] Wetzel C., Takeuchi T., Amano H. & Akasaki I. Quantized states in Ga_1-x_In_x_N/GaN heterostructures and the model of polarized homogeneous quantum wells. Phy. Rev. B 62, R12302–R13305 (2000).

[b8] Chichibu S. F. *et al.* Effective band gap inhomogeneity and piezoelectric field in InGaN/GaN multiquantum well structures. Appl. Phys. Lett. 73, 2006–2008 (1998).

[b9] Liu H. F. *et al.* Effects of annealing on structural and optical properties of InGaN/GaN multiple quantum wells at emission wavelength of 490 nm. J. Appl. Phys. 110, 063505 (2011).

[b10] O'Donnell K. P., Martin R. W. & Middleton P. G. Origin of Luminescence from InGaN Diodes. Phy. Rev. Lett. 82, 237–240 (1999).

[b11] Witzigmann B. *et al.* Microscopic analysis of optical gain in InGaN/GaN quantum wells. Appl. Phys. Lett. 88, 021104 (2006).

[b12] Chen X., Henderson B. & O'Donnell K. P. Luminescence decay in disordered low dimensional semiconductors. Apl. Phys. Lett. 60, 2672–2674 (1992).

[b13] Erol A., ed. (ed.), Dilute III-V Nitride Semiconductors and Material Systems: physics and technology (Springer, Berlin, Heidelberg, New York, 2008).

[b14] Pavesi L. & Ceschini M. Stretched-exponential decay of the luminescence in porous silicon. Phy. Rev. B 48, 17625–17628 (1993). 10.1103/physrevb.48.17625 10008389

[b15] Pophristic M., Long F. H., Tran C., Ferguson I. T. & Karlicek R. F. Jr Time resolved photoluminescence measurements of quantum dots in InGaN multiple quantum wells and light-emitting diodes. J. Appl. Phys. 86, 1114 (1999).

[b16] Li Z. Two distinct carrier localization in green light-emitting diodes with InGaN/GaN multiple quantum wells. J. Appl. Phys. 115, 083112 (2014).

[b17] Pozina G. *et al.* Origin of multiple peak photoluminescence in InGaN/GaN multiple quantum wells. J. Appl. Phys. 88, 2677 (2000).

[b18] Narukawa Y., Kawakami Y., Fujita S., Fugita S. & Nakamura S. Recombination dynamics of localized excitons in In_0.20_Ga_0.80_N-In_0.05_Ga_0.95_N multiple quantum wells. Phys. Rev. B 55, R1938–R1941 (1997).

[b19] Friel I., Thomidis C. & Moustakas T. D. Well width dependence of disorder effects on the optical properties of AlGaN/GaN quantum wells. Appl. Phys. Lett. 85, 3068–3070 (2004).

[b20] Reshchikov M. A. & Morkoç H. Luminescence properties of defects in GaN. Appl. Phys. Rev. 97, 061301 (2005).

[b21] Korotkov R. Y., Reshchikov M. A. & Wessels B. W. Acceptors in undoped GaN studied by transient photoluminescence. Physica B 325, 1–7 (2003).

[b22] Reshchikov M. A., Shahedipour F., Korotkov R. Y., Ulmer M. P. & Wessels B. W. Deep acceptors in undoped GaN. Physica B 273–274, 105–108 (1999).

[b23] Schmidt T., Lischka K. & Zulehner W. Excitation-power dependence of the near-band-edge photoluminescence of semiconductors. Phy. Rev. B 45, 8989–8994 (1992). 10.1103/physrevb.45.8989 10000759

[b24] Wang Q. Influence of annealing temperature on optical properties of InGaN quantum dot based light emitting diodes. Appl. Phys. Lett. 93, 081915 (2008).

